# Aerolysin-like proteins from *Armillaria ostoyae* with potential roles in plant pathogenicity reveal a distant evolutionary relationship to toadfish natterins

**DOI:** 10.3389/ffunb.2026.1894067

**Published:** 2026-07-21

**Authors:** Omar Languar, Simang Champramary, Boris Indic, Chetna Tyagi, Nóra Tünde Lange-Enyedi, Domokos Máthé, Tibor Kovács, Ferenc Budán, Bence Arnold Gucsik, Csaba Vágvölgyi, Younes Rezaee Danesh, László Kredics, György Sipos

**Affiliations:** 1 Functional Genomics and Bioinformatics Group, Faculty of Forestry, University of Sopron, Sopron, Hungary; 2 Department of Biotechnology and Microbiology, Faculty of Science and Informatics, University of Szeged, Szeged, Hungary; 3 Department of Biophysics and Radiation Biology, Semmelweis University, Budapest, Hungary; 4 Institute of Physiology, Medical School, University of Pécs, Pécs, Hungary; 5 Department of Plant Protection, Faculty of Agriculture, Van Yüzüncü Yil University, Van, Türkiye

**Keywords:** aerolysin-like proteins, *Armillaria ostoyae*, beta-pore-forming proteins, fungal pathogenicity, natterin, protein evolution

## Abstract

Understanding the evolutionary distribution and functional roles of toxins across diverse taxa remains a fundamental challenge in fungal biology. Aerolysin-like beta-pore-forming toxins are widely distributed across multiple kingdoms of life, yet their specific occurrence and structural diversity within the fungal kingdom remain poorly characterized. In our current study, we address this gap by investigating candidate aerolysin-like proteins in the basidiomycete *Armillaria ostoyae* using an integrated framework combining structural modeling, comparative genomics, and transcriptomic datasets spanning multiple developmental stages. Our results demonstrate that these candidate proteins are actively transcribed throughout the fungal life cycle, with consistent expression maintained in mature fruiting-body tissues. Notably, we show that the specific gene *ARMOST_18480* undergoes significant upregulation under plant-invasive conditions, strongly supporting its role as a putative pathogenicity-associated factor. Structural characterization revealed a modular architecture with deeply conserved pore-forming domains including Alanine-Glycine-Isoleucine-Proline (AGIP)-like loop variants homologous to vertebrate natterins from *Thalassophryne nattereri*, despite low overall sequence identity. Importantly, phylogenetic inference robustly resolves these *Armillaria* proteins within distinct fungal lineages well-separated from their vertebrate counterparts. Together, these findings significantly expand the known evolutionary distribution of the aerolysin superfamily and identify key candidates for future functional validation regarding pore-forming activity, plant pathogenicity, and mushroom-associated bioactivity.

## 1 Introduction

Species of the genus *Armillaria* are widely distributed wood-decaying basidiomycetes with important ecological roles in forest ecosystems as decomposers and plant pathogens (Sipos et al., 2017, 2018; Kedves et al., 2021). Several species, especially *Armillaria mellea* (honey fungus), are also valued as edible mushrooms and have attracted growing interest for their repertoire of bioactive compounds with putative medicinal properties (Erbiai et al., 2021). Yet despite their culinary use, retrospective epidemiological studies have documented occasional gastrointestinal symptoms associated with the consumption of *Armillaria* species, including nausea, vomiting, abdominal pain, and diarrhea, particularly following insufficient cooking or individual sensitivity (Cervellin et al., 2017; Gawlikowski et al., 2014; La Rosa et al., 2023). Similar reactions have also been reported for other edible mushroom species and are frequently associated with heat-labile or poorly characterized bioactive compounds (Benjamin, 1995; Wolf-Hall, 2014; Nieminen & Mustonen, 2020). However, the molecular basis underlying these reported biological effects remains poorly understood.

Fungi produce a broad range of extracellular or cell-surface localized proteins involved in ecological interactions, defense, and host colonization. Among these are pore-forming proteins, including members of the aerolysin superfamily, a group of β-pore-forming proteins characterized by conserved membrane interacting domains (Degiacomi et al., 2013). Originally described in bacteria such as *Aeromonas hydrophila*, members of this superfamily have since been identified across all kingdoms of life, including plants, protozoa, cnidarians, and fungi (Moran et al., 2012). Characterized fungal examples have previously been described, including Candidalysin from *Candida albicans* (Richardson et al., 2022; Ho et al., 2019) and aegerolysin-associated pore-forming proteins from *Aspergillus niger* and other fungal species (Kraševec, 2024). These examples highlight the structural and functional diversity of these fungal proteins.

A particularly intriguing group within the aerolysin superfamily is the natterin protein family, initially identified and characterized in the venom of the toadfish *T. nattereri*. Natterins (Natterin-1 through Natterin-4) are multifunctional toxins with kinin-releasing and nociceptive activities, defined by the conserved C-terminal aerolysin-like domain (Magalhaes et al., 2005; Lima et al., 2021).

Recent genomic studies have suggested that *Armillaria* species possess diverse repertoires of proteins and secondary metabolite-associated genes (Collins et al., 2017; Sipos et al., 2017; Caballero et al., 2022), including pore-forming proteins significantly expressed during fruiting body formation. However, little is known about the occurrence, diversity, and potential biological roles of the aerolysin-like proteins in *Armillaria* species.

This study therefore investigated the repertoire of candidate aerolysin-like proteins encoded in the genome of *A. ostoyae* strain C18/9. An integrated bioinformatic, genomic, transcriptomic, and structural analysis was performed using proteomics data to identify proteins containing aerolysin-like domains, evaluate their phylogenetic relationships, predict subcellular delivery pathways, and assess their structural similarity to known pore-forming proteins, including members of the natterin family.

## 2 Materials and methods

### 2.1 Phylogenetic analysis of aerolysin-like proteins

Initial BLASTp (https://blast.ncbi.nlm.nih.gov/blast/Blast.cgi) searches were performed to identify aerolysin-like homologs and trace their genetic diversities. These searches used the natterin protein sequences from the venomous toadfish *Thalassophryne nattereri* as queries against the genomes of *Armillaria*, *Desarmillaria*, and *Guayanagaster* species. In addition, to provide a broader evolutionary context for assessing the possible functional diversification levels within fungal eukaryotes, representative aerolysin-like proteins from various Basidiomycota and Ascomycota species were also included. Candidate sequences, confirmed by related proteomics data, exhibiting significant similarity to known aerolysin-like or natterin proteins were retained for further investigations.

For the pore-forming functionality-related phylogenetic reconstruction, only the conserved aerolysin-like domains were extracted from each protein sequence based on domain boundaries identified using the NCBI Conserved Domain Database (CDD) (Wang et al., 2022).

Then, before phylogenetic inference, multiple sequence alignments (MSAs) of the extracted domains were generated using Clustal Omega (EMBL) (https://www.ebi.ac.uk/jdispatcher/msa/clustalo?stype=protein). The resulting alignment was used to infer a maximum-likelihood phylogeny with IQ-TREE version 3.1.1 (Wong et al., 2025). The best-fitting amino acid substitution model was selected using ModelFinder implemented in IQ-TREE. Branch support was evaluated using the ultrafast bootstrap approximation (UFBoot) with 1,000 replicates. The final phylogenetic tree was visualized and annotated applying iTOL version 7.6 (https://itol.embl.de/).

At the end, the canonical bacterial aerolysin protein of *A. hydrophila* (PDB: 1PRE) was manually added as a reference sequence for the comparative analyses.

### 2.2 Identification and characterization of aerolysin-like proteins in *A. ostoyae*

Genome mining of candidate aerolysin-like proteins was initially performed using the genome of *A. ostoyae* strain C18/9 (NSCBI ID: GCA_900157425.1), and then the functional proteins were identified utilizing the proteomic datasets described by Sipos et al. (2017). Initial candidate genes were selected from prior transcriptomic annotations and predicted protein features consistent with toxin-like activity, including similarity to pore-forming proteins and the presence of conserved toxin-associated domains. The focus was on *A. ostoyae* as a primary pathogen, with high-quality gene expression datasets reflecting analyses of plant-invasive traits and of the developmental stages of fruiting body and rhizomorph formation already available (Sipos et al., 2017; Sahu et al., 2023).

To systematically identify aerolysin and aerolysin-like proteins, profile hidden Markov model (pHMM)-based searches were conducted using HMMER (hmmscan) (http://www.ebi.ac.uk/Tools/hmmer). Searches were performed against Pfam models (Paysan-Lafosse et al., 2024) corresponding to the aerolysin toxin domain (PF01117) and the aerolysin-like toxin domain (PF03318), using the reference proteomes databases (01/2025). Hits were retained based on Pfam gathering thresholds and E-value criteria. To detect more divergent homologs, iterative sequence-similarity searches were performed using JackHMMER. Domain assignments were further validated using batch conserved domain searches against the NCBI CDD (Wang et al., 2022).

These analyses identified four candidate aerolysin-like proteins in *A. ostoyae* (ARMOST_17075, ARMOST_18480, ARMOST_02021, and ARMOST_01910). Protein domain architecture was characterized for these candidates using the NCBI Batch CD-Search tool (Marchler-Bauer et al., 2010), and annotations were validated using both CDD and InterPro (Wang et al., 2022; Blum et al., 2024). Particular attention was given to aerolysin-like domains belonging to the PFM_aerolysin superfamily, as well as additional well-defined N-terminal regions such as ricin B-type lectin and fascin-like domains.

Based on domain conservation and annotation confidence, ARMOST_17075, ARMOST_18480, and ARMOST_02021 were selected for, whereas ARMOST_01910 was not included in subsequent downstream comparative analyses. Protein domain schematics were generated using IBS2.0 (Xie et al., 2022).

To identify homologous proteins in other related pathogenic *Armillaria* species, BLASTp (https://blast.ncbi.nlm.nih.gov/blast/Blast.cgi) searches were conducted against the available proteome of *A. mellea* obtained from the Joint Genome Institute (JGI) fungal genomics resource (Collins et al., 2013). Homologs were identified based on sequence identity, alignment coverage, and E-value thresholds.

Gene expression analysis of selected *A. ostoyae* candidate genes (ARMOST_17075, ARMOST_18480, and ARMOST_02021) was performed using RNA-seq datasets describing developmental stages of fungal growth (Sipos et al., 2017). To evaluate the potential role of candidate genes in pathogenicity, differential gene expression data from stem invasion assays were obtained from Sahu et al. (2023) and evaluated according to Indic and Sipos (2026). Data visualization was conducted in R version 4.6.1 using the ggplot2 package.

### 2.3 Subcellular localization, sequence analysis, and evolutionary characterization of aerolysin-like proteins

Subcellular localization and secretion potential of *A. ostoyae* candidate proteins (ARMOST_17075, ARMOST_18480, and ARMOST_02021) were assessed using multiple computational tools. Classical signal peptides were predicted using SignalP (Teufel et al., 2022). Transmembrane topology was evaluated with Phobius (Käll et al., 2004), and transmembrane helices were predicted with DeepTMHMM (Hallgren et al., 2022). With the help of DeepLoc 2.1 (Ødum et al., 2024), we predicted subcellular localization (Nielsen, 2025). Non-classical secretion pathways were evaluated using SecretomeP Proteins with scores above 0.6 were considered candidates for non-classical secretion (Bendtsen et al., 2004). In addition, OutCyte analysis was conducted for proteins lacking an N-terminal signal peptide and transmembrane helices to predict unconventional protein secretion (UPS), and proteins with scores above 0.5 were considered UPS candidates (Zhao et al., 2019).

MSA were performed to identify conserved regions and motifs. Initial alignments between full-length *A. ostoyae* proteins and natterin proteins from *T. nattereri* (Natterin-1 to Natterin-4) were conducted using Clustal Omega (https://www.ebi.ac.uk/jdispatcher/msa/clustalo). The resulting alignments were further inspected and visualized using Jalview version 2.1 (Waterhouse et al., 2009). Pairwise sequence alignments were performed using EMBOSS Needle (https://www.ebi.ac.uk/jdispatcher/psa/emboss_needle) to calculate sequence identity and similarity.

Selection pressure acting on natterin-like proteins was investigated using codon-based maximum likelihood analyses implemented in PAML (codeml) (Yang, 2007). Natterin-like sequences from the venomous fish *T. nattereri* and *A. ostoyae* were compared to evaluate evolutionary patterns and signatures of selection. Phylogenetic relationships were reconstructed using IQ-TREE with 1,000 bootstrap replicates. Protein sequences were aligned using Multiple Alignment using Fast Fourier Transform (MAFFT) (https://mafft.cbrc.jp/alignment/server/index.html), and codon-aware nucleotide alignments were generated using PAL2NAL (Suyama et al., 2006).

Several nested dN/dS (ω) models were applied in the Paml codeml. The M0 model assumed a single ω ratio across all branches, whereas the M2 branch model allowed separate ω ratios for fungal and fish lineages. Site models (M1a vs. M2a and M7 vs. M8) were used to test for positive selection acting on specific amino acid sites across all sequences. In addition, branch-site Model A was used to test for positive selection specifically affecting fungal branches. Pairwise dN/dS (Yang and Nielsen, 2000) estimates were also calculated using the (yn00) program as a robustness check. Values of ω < 1 were interpreted as evidence of purifying selection, ω = 1 as neutral evolution, and ω > 1 as positive selection.

Motif discovery was performed using the MEME (Multiple Em for Motif Elicitation) Suite version 5.5.9 (Bailey et al., 2006) to identify conserved sequence patterns across aerolysin-like proteins. Full-length protein sequences, including *A. ostoyae* candidate proteins, homologs from *A. mellea* and other fungal species, representative natterin proteins, and the canonical aerolysin structure (PDB: 1PRE), were used as input to avoid bias toward predefined regions. Motif analysis was conducted using the MEME algorithm in classic mode with the distribution set to “any number of repetitions per sequence” (anr). The maximum number of motifs was set to five, with motif widths ranging from 6 to 15 amino acids. Default background models and parameters were applied. The identified motifs were evaluated based on their statistical significance (E-values), sequence conservation, and distribution across taxa, with particular attention to motifs located within conserved pore-forming regions. ARMOST_01910 was excluded from motif discovery analysis due to its high sequence divergence and incomplete representation of conserved aerolysin-like regions, which could bias motif detection and reduce the reliability of identified conserved patterns.

Three-dimensional (3D) protein structures of ARMOST_18480, ARMOST_17075, and ARMOST_02021 were predicted using the AlphaFold3 server (https://alphafoldserver.com/). The resulting structural models were exported in PDB format and included as **Supplementary Materials**.

## 3 Results

### 3.1 Phylogenetic relationships of aerolysin-like proteins

**Figure 1:**
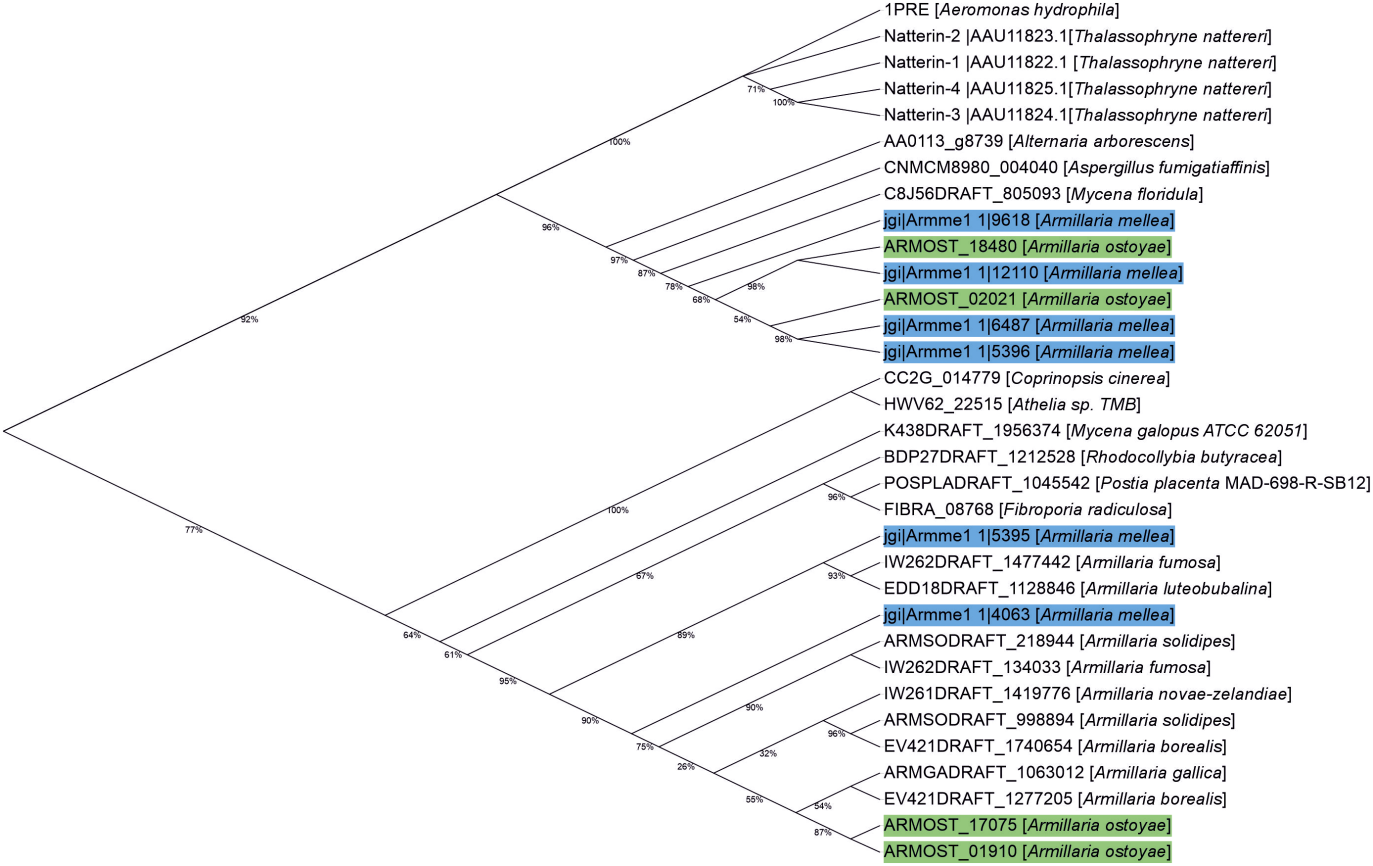
Phylogenetic analysis of aerolysin-like domains. Proteins from *A. ostoyae* and *A. mellea* are highlighted in green and blue, respectively.

The maximum likelihood phylogenetic analysis revealed a clear and well-supported separation between natterin proteins from the venomous fish *T. nattereri* (Natterin-1 to Natterin-4) and all fungal sequences included in the dataset (**Figure 1**). The Natterin proteins formed a distinct monophyletic clade, reflecting their close evolutionary relationship and confirming their divergence from fungal aerolysin-like homologs. All fungal sequences clustered within a separate, well-defined clade. Within this group, Ascomycota representatives, including *Alternaria arborescens* and *Aspergillus fumigatiaffinis*, together with *Mycena floridula*, were positioned outside the main *Armillaria*-dominated clusters, indicating their phylogenetic separation from the core Basidiomycota lineage. In contrast, the majority of sequences, including *Armillaria* species, *Coprinopsis cinerea*, *Athelia* sp., *Mycena galopus*, *Postia placenta*, and *Fibroporia radiculosa*, formed a cohesive cluster within the Basidiomycota.

The four *A. ostoyae* proteins were consistently placed within the fungal clade and remained clearly distinct from the Natterin lineage. ARMOST_02021 and ARMOST_18480 grouped within a well-supported subcluster together with *A. mellea* homologs (Armme1_1|6487, Armme1_1|9618, and Armme1_1|12110), indicating close evolutionary conservation between homologous proteins of these species. In contrast, ARMOST_17075 and ARMOST_01910 were resolved in a separate subcluster together with homologs from other *Armillaria* species, including *A. borealis*, *A. gallica*, *A. solidipes*, and *A. fumosa*, as well as the *A. mellea* protein Armme1_1|4063. This distinct phylogenetic placement suggests functional or evolutionary divergence of this protein relative to the other *A. ostoyae* homologs. Overall, the maximum likelihood topology supports the classification of *A. ostoya*e proteins as members of the aerolysin-like superfamily while clearly delineating them from vertebrate natterins. The observed clustering patterns indicate lineage-specific diversification within *Armillaria* and broader fungal taxa, consistent with the evolutionary expansion and functional adaptation of aerolysin-like proteins across fungi.

### 3.2 Identification of homologous aerolysin-like proteins in *Armillaria mellea*

BLAST-based homology searches identified three high-confidence homologs corresponding to the selected *A. ostoyae* candidate proteins: Armme1_1|4063 (87.4% identity for ARMOST_17075), Armme1_1|12110 (78.3% identity for ARMOST_18480), and Armme1_1|6487 (64.8% identity for ARMOST_02021) (**Supplementary Table 1**). All three homologs share conserved InterPro annotations, including the natterin C-terminal domain (IPR053237) and aerolysin-like toxin domain (IPR004991), supporting their classification within the aerolysin/natterin toxin family.

### 3.3 Subcellular localization and potential extracellular delivery of aerolysin-like proteins

SignalP analysis indicated that none of the analyzed proteins, including the *A. ostoyae* candidates and their *A. mellea* homologs, contain classical signal peptides. Consistently, Phobius predicted all proteins as non-cytoplasmic. DeepTMHMM analysis further showed that all proteins lack transmembrane helices (0 TMHs), indicating that they are not membrane-anchored (**Supplementary Table 2**). DeepLoc 2.1 predicted extracellular localization for the majority of proteins in both species. Specifically, all *A. mellea* homologs and three *A. ostoyae* proteins (ARMOST_17075, ARMOST_18480, and ARMOST_02021) were predicted to be extracellular and soluble (**Supplementary Table 2**). SecretomeP scores ranged from 0.272 to 0.608. Only Armme1_1|6487 (0.608) exceeded the threshold (≥0.6), suggesting potential non-classical secretion (**Supplementary Table 2**).

Outcyte analysis was performed to further evaluate the unconventional secretion potential of the identified *A. ostoyae* aerolysin-like proteins ARMOST_17075, ARMOST_18480, and ARMOST_02021 were all predicted as UPS candidates, with OutCyte scores of 0.5611, 0.6368, and 0.6368, respectively. Among these, ARMOST_18480 and ARMOST_02021 exhibited the highest scores, supporting their potential extracellular secretion via non-classical pathways.

### 3.4 Genome-wide identification and domain architecture of aerolysin-like proteins in *Armillaria ostoyae*

Profile hidden Markov model-based screening using HMMER identified a limited set of aerolysin-related proteins in the *A. ostoyae* proteome. Using the aerolysin-like toxin profile, three candidate proteins were detected: ARMOST_17075, ARMOST_18480, and ARMOST_02021 (**Supplementary Table 3**). Among these, ARMOST_17075 exhibited the strongest statistical support (E-value = 1.4 × 10^-10^), followed by ARMOST_18480 (E-value = 9.0 × 10^-8^), whereas ARMOST_02021 displayed weaker support (E-value = 1.3 × 10^-2^), indicative of a more divergent sequence. Application of the canonical aerolysin toxin profile resulted in the detection of only a single protein, ARMOST_17075 (E-value = 4.6 × 10^-3^), suggesting that this protein represents the closest homolog to classical aerolysin pore-forming toxins within *A. ostoyae*. The absence of additional hits using this profile indicates that the remaining proteins are more evolutionarily divergent and are not captured by the canonical toxin model.

Iterative sequence similarity searches using JackHMMER further supported these findings. Using ARMOST_17075 as the query, the three proteins ARMOST_01910, ARMOST_18480 and ARMOST_02021 were consistently recovered with strong statistical support (E-values ranging from 8.4 × 10^-27^ to 1.8 × 10^-8^) respectively. In addition, ARMOST_01910 (E-value = 8.4 × 10^-27^) was identified, representing a putative homolog not detected in the initial domain-based screening (**Supplementary Table 3**). However, ARMOST_01910 was excluded from subsequent comparative and structural analyses due to its weak domain conservation and lower confidence as a representative aerolysin-like protein.

Conserved domain analysis further confirmed that the three primary candidate *A. ostoyae* proteins contain aerolysin-like domains belonging to the PFM_aerolysin superfamily (cl40431), with highly significant E-values ranging from 1.1716 × 10^-92^ to 1.11073 × 10^-74^ (**Supplementary Table 4**).

**Figure 2:**
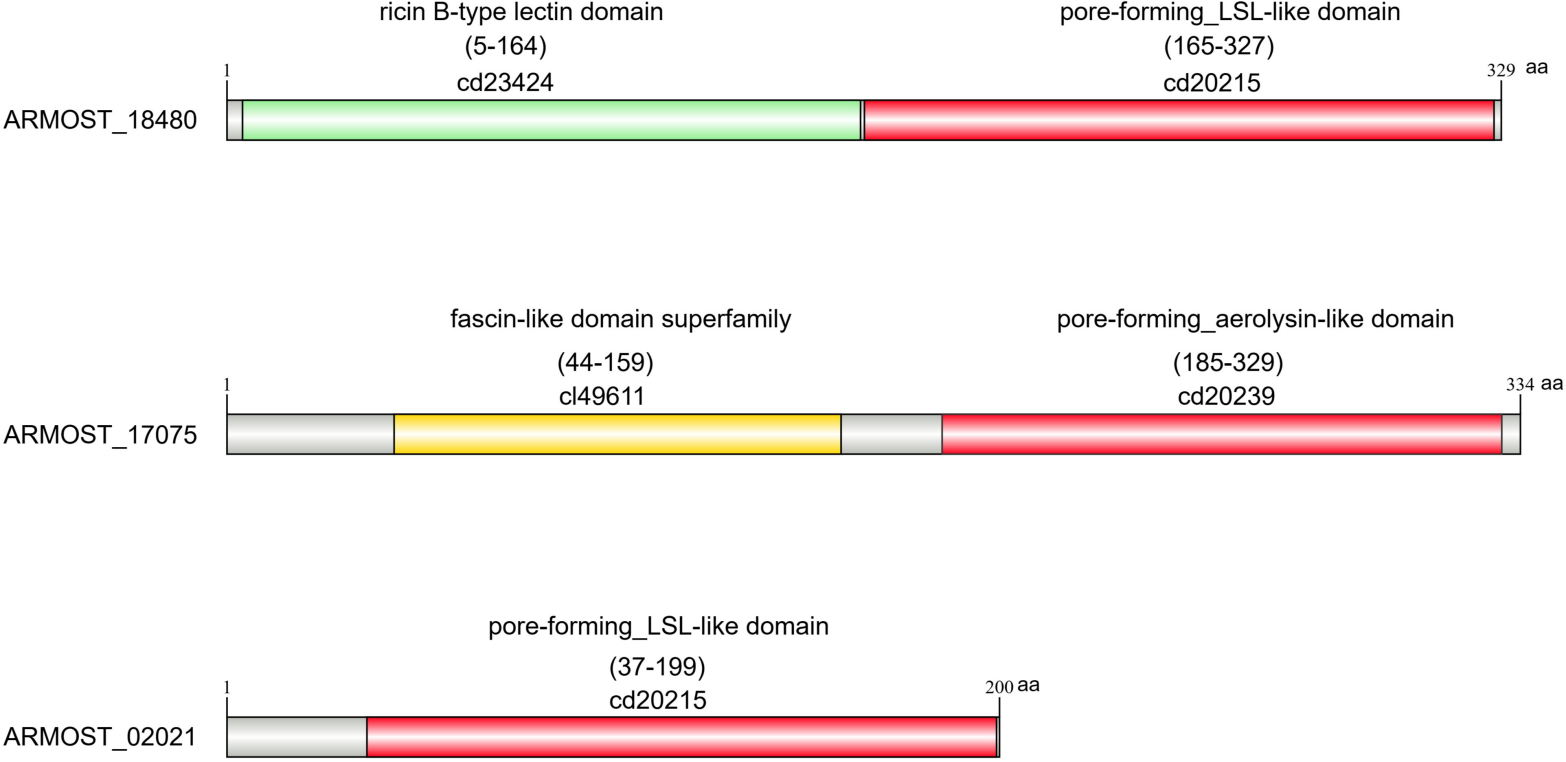
Domain organization of aerolysin-like proteins in *A. ostoyae* identified by conserved domain analysis (IDs indicated at the specific domains). Predicted domains include N-terminal regions: ricin B-type lectin domains (green) and fascin-like domain superfamily (yellow), and C-terminal aerolysin-like domains (red). Despite variation in domain annotations, all proteins exhibit a conserved aerolysin-like architecture.

As shown in **Figure 2**, the three proteins display distinct domain architectures. ARMOST_18480 contains an N-terminal ricin B-type lectin domain (cd23424) coupled with a C-terminal LSL-like pore-forming domain (cd20215). ARMOST_17075 exhibits a fascin-like domain (cl49611) linked to a C-terminal aerolysin-like domain (cd20239). In contrast, ARMOST_02021 lacks an identifiable N-terminal domain and consists primarily of an LSL-like pore-forming module (cd20215). **Supplementary Figure 1** shows the domain organization of the *A. ostoyae* proteins including additional *A. mellea* proteins sharing similar architectures. Overall, these proteins are characterized by conserved pore-forming domains (LSL-like or aerolysin-like) combined with variable N-terminal regions, including ricin B-type lectin or fascin-like domains. While ARMOST_18480 and ARMOST_17075 contain additional domains, ARMOST_02021 is largely composed of the pore-forming domain, highlighting structural variability within this protein group.

### 3.5 Transcriptional profiling of aerolysin-like genes across developmental stages and plant-invasive conditions

**Figure 3:**
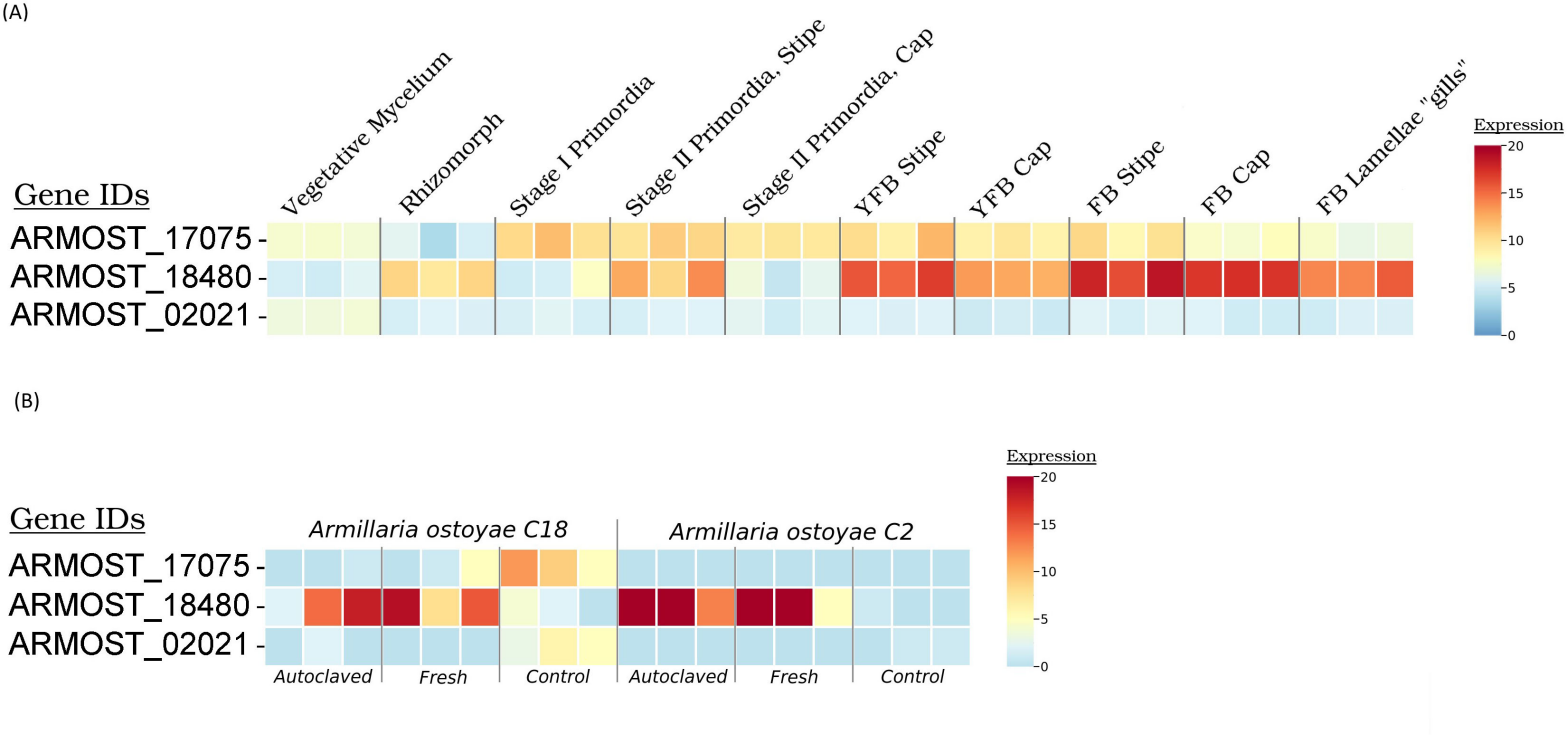
Expression profile of candidate aerolysin-like genes in *A. ostoyae* across different developmental stages and environmental conditions. **(A)** Gene expression across developmental stages, including vegetative mycelium, rhizomorphs, primordia, and fruiting body tissues. **(B)** Gene expression under different conditions (control, autoclaved, and fresh substrate) in high-virulent C18 and low-virulent C2 strains.

Across developmental stages (**Figure 3A**), all three genes were expressed throughout the life cycle, but with notable differences in expression levels and tissue specificity. ARMOST_18480 showed the highest overall expression, with strong upregulation in fruiting body tissues, including cap, stipe, and lamellae, as well as in young fruiting structures. ARMOST_17075 exhibited moderate expression, with increased levels observed during primordia and early developmental stages, followed by variable expression in fruiting tissues. In contrast, ARMOST_02021 displayed consistently low expression across all stages, with only minor variation between tissues.

To further evaluate expression under different conditions, transcription levels were compared between two strains with distinct virulence levels (C18 - high and C2 - low) under autoclaved, fresh, and control conditions (**Figure 3B**). Focusing on the control condition, which reflects baseline expression, ARMOST_18480 showed the highest expression in both strains again, indicating consistent transcription under normal conditions. ARMOST_17075 exhibited moderate expression, while ARMOST_02021 remained lowly expressed.

Under autoclaved and fresh conditions, significant differential expression profiles were observed for all three genes. ARMOST_18480 maintained relatively high expression levels across both strains, whereas ARMOST_17075 showed moderate variability under different conditions. ARMOST_02021 displayed minimal changes and remained consistently lowly expressed. Although some differences between the two strains were observed across conditions, all three genes were expressed in both C18 and C2, indicating that their transcription is not strain-specific.

Based on its expression under invasive conditions, ARMOST_18480 may therefore represent a candidate pathogenicity factor.

### 3.6 Sequence conservation, motif architecture, and selection pressure of aerolysin-like proteins

**Figure 4:**
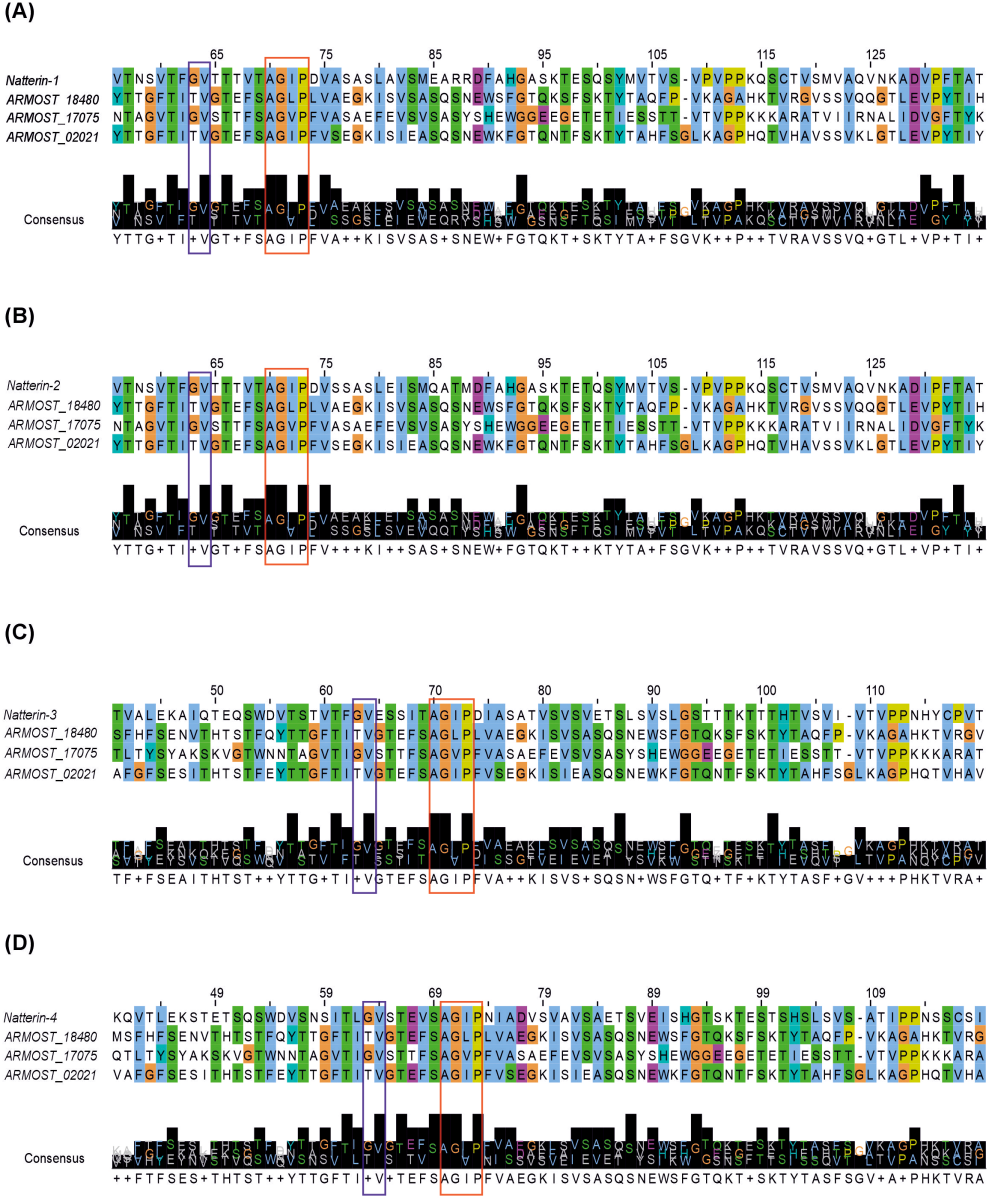
Alignment of *A. ostoyae* aerolysin-like domains with conserved domains of Natterin proteins (Natterin-1 to -4). **(A)** Region of *A. ostoyae* proteins aligned with the Natterin-1 conserved domain. **(B)** Region of *A. ostoyae* proteins aligned with the Natterin-2 conserved domain. **(C)** Region of *A. ostoyae* proteins aligned with the Natterin-3 conserved domain. **(D)** Region of *A. ostoyae* proteins aligned with the Natterin-4 conserved domain. Conserved AGIP-like motifs are highlighted in red, while conserved GV residues are highlighted in blue.

To identify conserved regions, multiple sequence alignments were performed between full-length *A. ostoyae* proteins and Natterin-1 to Natterin-4. These alignments were used to define the regions corresponding to the aerolysin-like domains. The identified regions were subsequently extracted and re-aligned with the conserved domains of natterin proteins (**Figures 4A–D**).

The alignment confirmed that all *A. ostoyae* proteins correspond to the aerolysin-like regions of natterins, supporting their structural homology. A conserved AGIP motif was consistently observed in natterin proteins, while *A. ostoyae* proteins showed variant forms of this motif: ARMOST_18480 contains an AGLP motif, ARMOST_17075 an AGVP motif, and ARMOST_02021 retains the canonical AGIP motif.

Pairwise sequence alignment of these domains against Natterin proteins (Natterin-1 to Natterin-4), performed using EMBOSS Needle, revealed low sequence identity and moderate similarity. Identity values ranged from 15.3% to 29.8%, while similarity ranged from 31.2% to 48.0% (**Supplementary Table 5**). Among the analyzed proteins, ARMOST_17075 showed the highest overall similarity to Natterins, followed by ARMOST_18480, while ARMOST_02021 displayed the lowest similarity across all comparisons.

**Figure 5:**
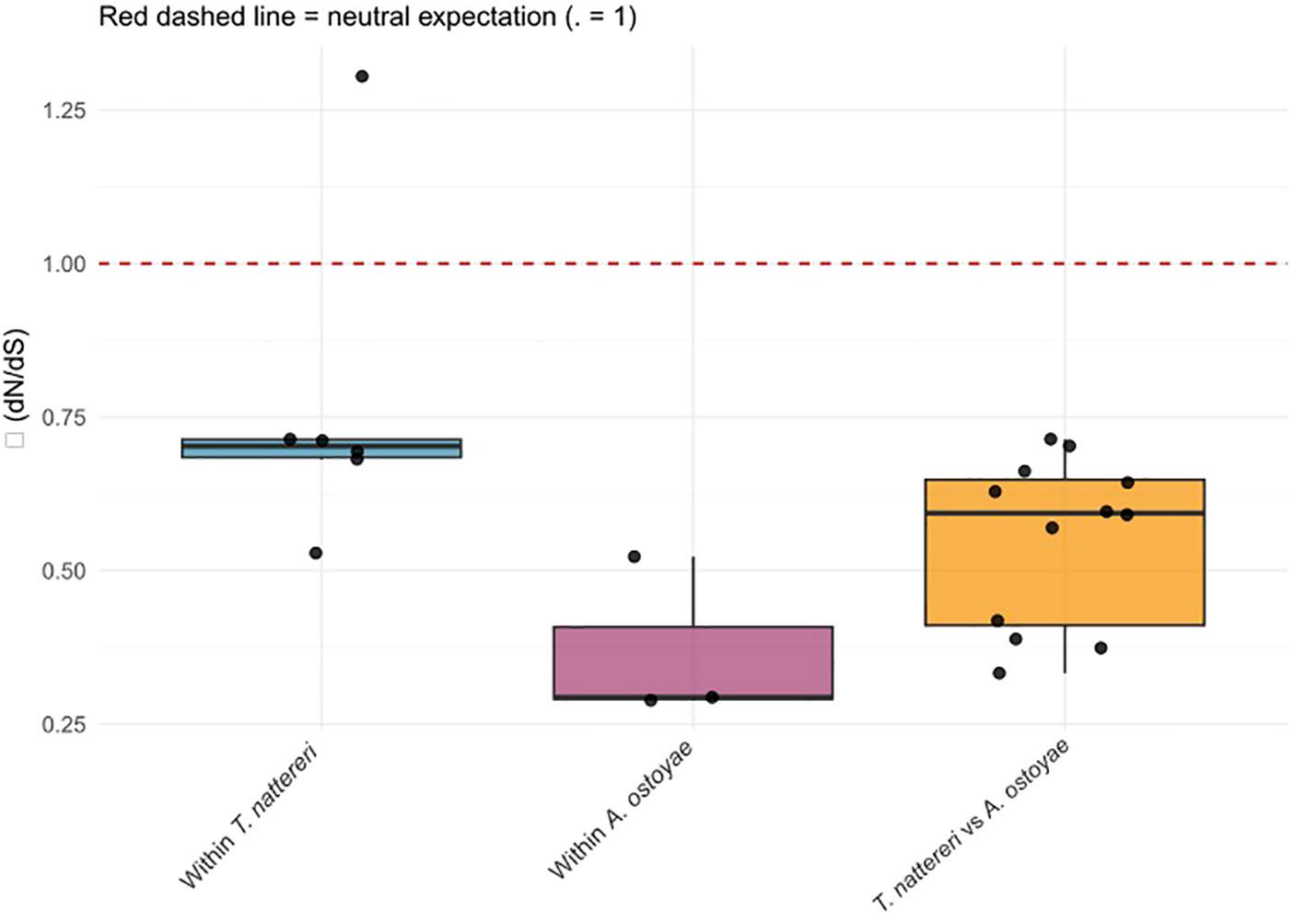
Pairwise dN/dS ratios between *T. nattereri* and *A. ostoyae* proteins.

Codon-based evolutionary analyses showed that the analyzed proteins are predominantly evolving under purifying selection. The single-ratio model (M0) estimated an overall ω (dN/dS) value of 0.428. Branch-model analyses showed lower ω values in *A. ostoyae* (ω = 0.300) than in *T. nattereri* (ω = 0.504), indicating stronger evolutionary constraint in the fungal lineage. Site-model comparisons (M1a vs. M2a and M7 vs. M8) were not statistically significant (p = 1.000 and p = 0.678, respectively). However, branch-site Model A detected significant positive (ω = 1) selection in the fungal lineage (2ΔlnL = 6.45, p = 0.011). Pairwise (yn00) analyses yielded ω values of 0.29-0.52 within *A. ostoyae*, 0.53-1.3 within *T. nattereri*, and 0.33-0.71 between fungal and fish proteins (**Figure 5**; **Supplementary Table 6**). The elevated upper bound in *T. nattereri* was driven by Natterin-1 and Natterin-2 (ω ~ 1.3). Phylogenetic reconstruction separated fungal and fish proteins into distinct clades (**Supplementary Figure 2**).

*De novo* motif discovery using MEME Suite identified five conserved motifs across the analyzed aerolysin-like protein sequences (**Supplementary Table 7**). All motifs exhibited strong statistical support, with E-values ranging from 7.7 × 10^-117^ to 6.4 × 10^-257^. Among these, Motif 2 (E-value = 2.4 × 10^-151^) showed the broadest distribution across the dataset. The consensus sequence of this motif (TFSAGVPFVAS) contains an AGIP/AGVP-like core and was detected in all analyzed *A. ostoyae* proteins, as well as in natterins and other fungal homologs.

*In A. ostoyae*, this motif is present as variant forms, including AGVP (ARMOST_17075), AGLP (ARMOST_18480), and AGIP (ARMOST_02021). The motif occurs within a conserved sequence context across the analyzed proteins. The remaining motifs also showed strong statistical support, including Motif 1 (E-value = 6.4 × 10^-257^), Motif 3 (2.0 × 10^-143^), Motif 4 (2.5 × 10^-136^), and Motif 5 (7.7 × 10^-117^). The motifs are presented in **Supplementary Figure 3**, and the same Motif 2 was also detected in the canonical aerolysin structure (PDB ID: 1PRE).

### 3.7 Structural organization and AlphaFold-based modeling of aerolysin-like proteins

**Figure 6:**
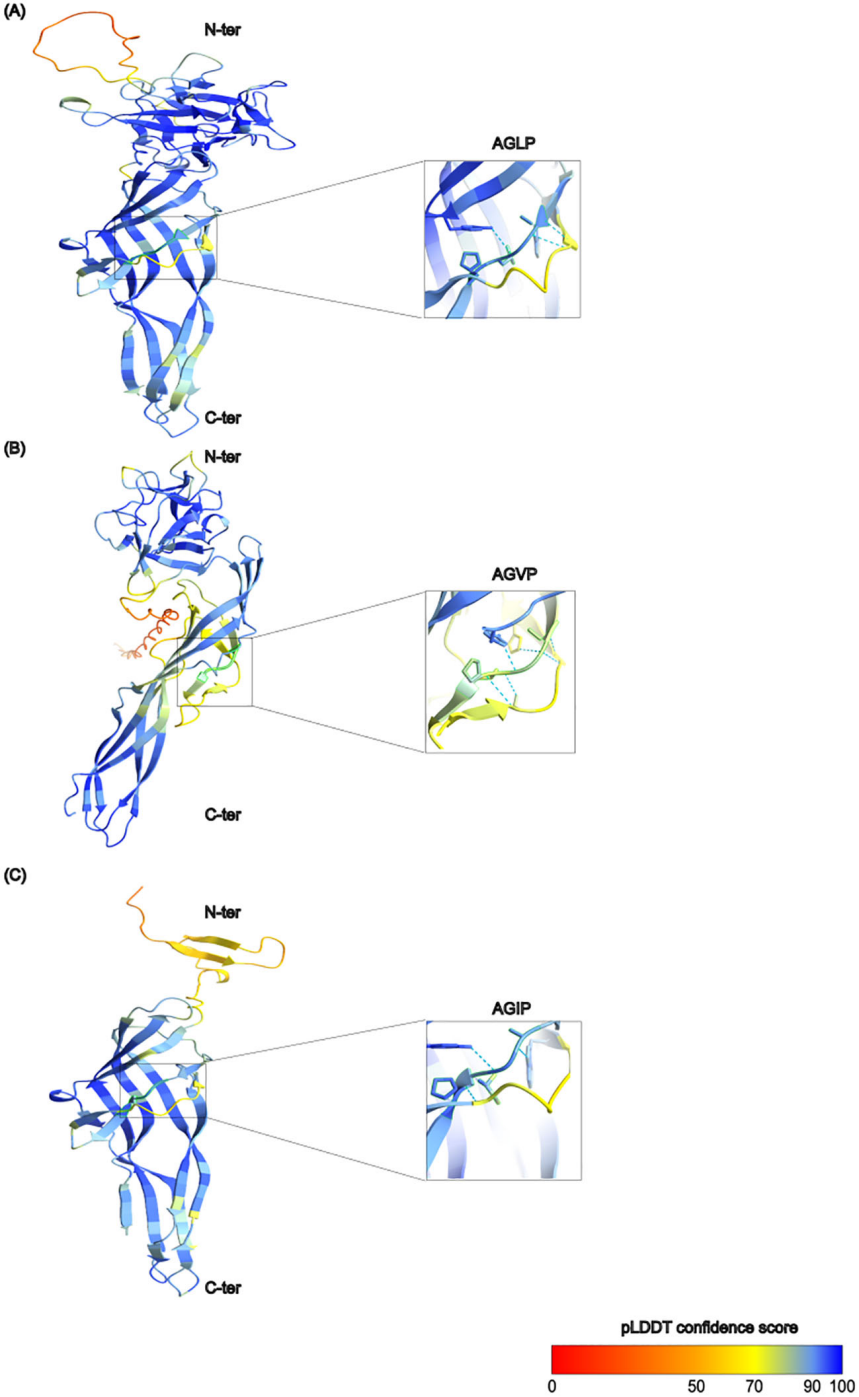
Predicted structures of *A. ostoyae* aerolysin-like proteins. **(A)** ARMOST_18480, **(B)** ARMOST_17075, and **(C)** ARMOST_02021. Conserved AGIP-like motifs (AGLP, AGVP, and AGIP) located within the predicted pore-forming regions are highlighted and shown as enlarged views. pLDDT refers to predicted local distance difference test.

Domain organization revealed that all three *A. ostoyae* proteins share a modular organization consisting of an N-terminal domain and a C-terminal region (**Figure 2**). Specifically, ARMOST_18480 contains a ricin B-type lectin domain, whereas ARMOST_17075 contains a fascin-like domain. In contrast, ARMOST_02021 lacks a defined N-terminal accessory domain and is primarily composed of a pore-forming region. Despite differences in N-terminal domain composition, all proteins exhibit a similar overall structural arrangement, with the N-terminal region positioned upstream of the C-terminal domain. Predicted 3D structures generated using AlphaFold3 further supported this organization, showing consistent folding patterns across all three proteins (**Figure 6**). The models revealed well-defined structural regions corresponding to both the N-terminal and C-terminal parts of the proteins. The identified motifs (AGLP, AGVP, and AGIP) are highlighted in red in the structural models, indicating their spatial localization within the predicted pore-forming regions.

Model confidence analysis revealed differences in structural reliability among the three proteins. Predicted local distance difference test (pLDDT) evaluates confidence in the local structure by estimating how closely the predicted model would match an experimentally determined structure. The AlphaFold-predicted structure of ARMOST_18480 exhibited overall high confidence, with most residues showing pLDDT scores above 90, indicating a well-defined structural core, while only limited regions displayed moderate confidence. In contrast, ARMOST_02021 showed variable confidence, with low pLDDT values (<50) in the N-terminal region and moderate to high confidence scores (70-90) across the majority of the structure, suggesting a stable core with flexible regions. Similarly, ARMOST_17075 displayed variable confidence, with low-confidence regions in the N-terminus and higher confidence (>80) across the main structural core.

The predicted TM-scores (pTM) evaluate the accuracy of the complex’s overall structural arrangement. pTM further supported overall model reliability, with values of 0.82 for ARMOST_18480, 0.77 for ARMOST_02021, and 0.69 for ARMOST_17075, indicating moderate to high confidence in the global fold of the predicted structures. Predicted aligned error (PAE) analysis showed generally low error across most regions, with localized areas of increased uncertainty, particularly in flexible or less conserved regions (**Supplementary Figure 4**).

Structural comparison revealed limited similarity to the canonical aerolysin structure (PDB: 1PRE), with low TM-scores ranging from 0.25 - 0.31. In contrast, higher TM-scores (0.40-0.50) were observed when compared to natterin proteins, indicating closer structural similarity to this group despite low overall sequence identity (**Supplementary Table 8**).

## 4 Discussion

Aerolysin-like proteins are a conserved family of β-pore-forming toxins (β-PFTs) identified across multiple kingdoms of life, including the prototypical aerolysin produced by *Aeromonas* species and the fungal lectin LSL from the bracket fungus *Laetiporus sulphureus*, highlighting their broad evolutionary distribution and functional conservation (Cirauqui et al., 2017; Gurnev and Nestorovich, 2014).

Phylogenetic analysis based on the conserved aerolysin domain using a maximum-likelihood approach revealed a clear separation between natterin proteins and fungal sequences, with both groups forming distinct, well-supported clades (**Figure 1**). This pattern is consistent with the evolutionary distribution of the aerolysin superfamily across distantly related taxa (Moran et al., 2012). Within fungi, *Armillaria* proteins, including *A. ostoyae* and *A. mellea* homologs, clustered within the Basidiomycota lineage while forming distinct subgroups, indicating conservation within the genus alongside evidence of diversification. In particular, the separation of ARMOST_17075 from the other *A. ostoyae* homologs suggests functional or evolutionary divergence within this protein family.

This subdivision is consistent with the diversity of aerolysin-like proteins reported in fungi, where multiple distinct groups are observed within the same lineage (Szczesny et al., 2011). Despite their clear phylogenetic separation from vertebrate natterins, the conservation of aerolysin-like domains supports their classification within the same broader superfamily. Overall, the observed clustering patterns support the view that aerolysin-like proteins in *A. ostoyae* and *A. mellea* represent conserved yet diversified members of the aerolysin superfamily, maintaining structural features associated with pore-forming activity as described for the canonical aerolysin fold represented by the 1PRE used as a reference structure (Moran et al., 2012).

The high sequence similarity (64.8% to 87.4%) between *A. ostoyae* and *A. mellea* proteins indicates strong conservation of aerolysin-like domains, consistent with shared evolutionary ancestry. Conserved domain architecture suggests structural constraints linked to functional preservation, while variation in sequence identity likely reflects divergence within a common fold. Such patterns are typical of homologous domain families, where structure is maintained despite sequence evolution (Ponting and Russell, 2002).

The aerolysin-like proteins in *A. ostoyae* and *A. mellea* are predominantly predicted to be soluble and extracellular despite lacking classical signal peptides, suggesting possible secretion via non-classical mechanisms. Although DeepLoc predicted extracellular localization for these proteins, the absence of detectable signal peptides indicates that extracellular delivery may occur through unconventional secretion rather than the classical ER-Golgi route. In eukaryotes, protein secretion occurs either through the classical pathway, which relies on N-terminal signal peptides and ER-Golgi trafficking, or through unconventional protein secretion (UPS), which bypasses the Golgi apparatus and may involve extracellular vesicles, direct translocation across the plasma membrane, or autophagy-associated mechanisms (Ding et al., 2012). This is supported by SecretomeP scores above the threshold (>0.6) for Armme1_1|6487 (Bendtsen et al., 2004). The absence of transmembrane domains further indicates that these proteins are not membrane-bound. However, OutCyte scores above 0.5 for ARMOST_17075 (0.5611), ARMOST_18480 (0.63688), and ARMOST_02021 (0.6368) place all three proteins within the range of credible UPS candidates. Whether these proteins are secreted constitutively or in a condition-dependent manner remains to be established experimentally; the expression data suggest that at least ARMOST_18480 is available for secretion during both developmental transitions and plant invasion, but direct biochemical evidence for its extracellular localization is lacking and constitutes an important priority for future work.

In this study, we structurally characterized three aerolysin-like proteins in *A. ostoyae*. Domain analysis showed that all three proteins harbor pore-forming regions associated with the PFM_aerolysin superfamily, a group of β-pore-forming toxins widely distributed across different biological kingdoms (Szczesny et al., 2011). ARMOST_18480 and ARMOST_02021 contain LSL-like domains (cd20215), whereas ARMOST_17075 carries an aerolysin-like domain (cd20239). In contrast to ARMOST_18480, which includes an additional N-terminal lectin domain, ARMOST_02021 is largely composed of the pore-forming region and lacks a distinct N-terminal accessory domain, indicating a more compact architecture. ARMOST_01910 was excluded due to its low-confidence domain hits.

The LSL-like domains identified in ARMOST_18480 and ARMOST_02021 are related to the hemolytic lectin LSL described in *L. sulphureus* (Mancheño et al., 2005), while the aerolysin-like domain in ARMOST_17075 belongs to the same broader aerolysin superfamily, supporting the presence of a conserved pore-forming core among all three proteins (Podobnik et al., 2017).

Consistent with this conserved pore-forming architecture, variation is observed in the N-terminal regions, supporting a modular organization of these proteins. ARMOST_18480 contains a ricin B-type lectin domain, and similar domains are present in its *A. mellea* homologs Armme1_1|12110 and Armme1_1|6487. These ricin B-type lectin (beta-trefoil) domains are known to mediate carbohydrate binding, suggesting a potential role in target recognition (Aragão et al., 2008; Rini, 1995). Notably, many lectins exhibit remarkable structural stability, retaining activity under conditions that typically denature proteins. For instance, several plant and animal lectins remain stable after exposure to temperatures around 70 °C for more than 30 min and show resistance to proteolytic enzymes and acidic environments. This stability allows a proportion of lectins to persist during digestion, and experimental observations indicate that approximately 1-5% may be absorbed across the intestinal barrier into the bloodstream in animals, where they can contribute to physiological or immune responses (Kumar et al., 2012).

In microbial systems, lectins and lectin-like proteins have been shown to interact with cell surface polysaccharides such as mannans and other glycoconjugates, highlighting their role in host-microbe interactions and surface recognition (Pistole, 1981).

Proteins adopting a β-trefoil fold have been associated with diverse functional roles, including protease inhibition in fungi. For example, *A. mellea* and other basidiomycetes have been reported to produce thermostable trypsin inhibitors (Lukanc et al., 1970). Structurally, β-trefoil proteins share a conserved β-strand core, while functional specificity is mediated by variable loop regions (Blaber, 2022). In our study, ARMOST_18480 contains an N-terminal ricin B-type lectin domain predicted to adopt a β-trefoil structure, consistent with *A. mellea* homologs (Armme1_1|12110 and Armme1_1|6487), as well as related proteins such as Armme1_1|9618 (**Supplementary Figure 1**).

By contrast, ARMOST_17075 displays a different domain organization, with an N-terminal fascin-like domain linked to a C-terminal aerolysin-like region. A similar architecture is observed in Armme1_1|4063. Such modular arrangements, combining additional domains with pore-forming regions, have been described for aerolysin-like proteins (Dang et al., 2017). Because this annotation is only domain-based, any actin-related function would require experimental validation.

Gene expression profiling showed that the identified aerolysin-like genes are actively transcribed across the life cycle of *A. ostoyae*, with distinct expression levels of the candidates. The strong expression of ARMOST_18480 in fruiting bodies raises the possibility that aerolysin-like proteins may contribute to the bioactive profile of *Armillaria* fruiting bodies. Although pore-forming toxins were previously classified among defense-related genes in *Armillaria* (Sipos et al., 2017), our expression analysis under invasive conditions suggests a different functional role for ARMOST_18480. The elevated expression of ARMOST_18480 during host-invasive conditions is particularly noteworthy, as proteins that interact with or disrupt cellular membranes have frequently been associated with fungal pathogenicity. For example, necrosis- and ethylene-inducing-like proteins (NLPs), which are widespread among plant-associated microorganisms, can induce membrane damage and cell death in susceptible hosts, thereby contributing to disease development and host colonization (Pirc et al., 2022). Similarly, pore-forming proteins have been implicated in microbial virulence through their ability to alter membrane integrity and mediate interactions with host cells (Peraro and Van Der Goot, 2015). Although no direct evidence currently demonstrates that ARMOST_18480 possesses pore-forming activity during plant infection, its predicted aerolysin-like domain architecture, extracellular localization, and increased expression under invasive conditions collectively suggest a potential role in host-pathogen interactions. Nevertheless, these observations remain correlative, and experimental studies, including gene knockout approaches, heterologous expression, and membrane activity assays, will be required to determine whether ARMOST_18480 contributes directly to pathogenicity in *A. ostoyae*. In contrast, the consistently low expression of ARMOST_02021, which encodes only an aerolysin structure without a targeting domain, across all tested conditions suggests either tight regulation or a context-dependent role not captured by the available datasets.

Comparative alignment of *A. ostoyae* proteins with natterin conserved domains revealed partial conservation of key sequence motifs within the aerolysin-like region. As reported by Lima et al. (2021), natterin proteins consistently contain a conserved AGIP motif within the pore-forming loop of the aerolysin domain. In agreement with this, ARMOST_02021 retains the canonical AGIP motif, whereas variant forms were identified in the other proteins, including AGVP in ARMOST_17075 and AGLP in ARMOST_18480, indicating that the AGIP motif is conserved but modified within *A. ostoyae* proteins. In addition to the AGIP motif, *de novo* motif analysis identified a highly conserved motif (Motif 2: TFSAGVPFVAS) present across all *A. ostoyae* proteins as well as natterins, 1PRE, and other fungal homologs. This motif contains an AGIP/AGVP-like core and occurs within a conserved sequence context, further supporting its structural conservation. Hence, the identified motif in *A. ostoyae* may be associated with structural and functional roles similar to those reported for Natterin-like proteins. Lima et al. (2021) described this conserved motif as part of the pore-forming region involved in membrane interaction and structural stabilization. The variability observed among *Armillaria* motifs, including AGLP and AGVP, may reflect evolutionary adaptations that could influence protein function and interaction dynamics while maintaining the conserved core characteristics of the motif.

Pairwise sequence alignment of the *A. ostoyae* aerolysin-like domains against the conserved domains of natterin proteins revealed low sequence identity (15.3-29.8%) but moderate similarity (31.2-48.0%) (**Supplementary Table 5**). These values fall within the range described as the “twilight zone” of sequence alignment, where sequence identity below ~30% limits the reliability of homology detection using standard sequence-based approaches (Rost, 1999). At this level of divergence, functional and structural relationships may not be readily apparent from primary sequence comparisons alone. Notably, this conserved motif was also detected in the canonical aerolysin structure, further supporting its association with structurally conserved regions of pore-forming toxins.

Nevertheless, proteins with low sequence identity can retain conserved structural features, particularly when key residues or motifs are preserved. Structural studies have shown that homologous proteins may maintain similar folds despite substantial sequence divergence (Blake and Cohen, 2001). This pattern is consistent with aerolysin-like proteins, which are broadly distributed across different kingdoms and often display low overall sequence similarity while preserving functionally relevant residues. Moran et al. (2012) reported that many aerolysin homologues share less than ~20% sequence similarity but retain conserved residues associated with pore-forming activity, frequently embedded within short, conserved motifs.

The predominance of ω values below 1 suggests that these proteins are mainly evolving under purifying selection, consistent with the conservation of functionally important aerolysin-domain proteins (Yang and Bielawski, 2000; Anderluh and Lakey, 2010; Dasmeh et al., 2014). Lower ω values in *A. ostoyae* compared to *T. nattereri* further indicate stronger evolutionary constraint in the fungal lineage.

Although site-model analyses did not detect evidence of positive selection across all sequences, the significant branch-site result suggests that adaptive evolution may have affected specific codons within the fungal lineage (Zhang, 2005; Yang and Reis, 2010). In contrast, the comparatively higher ω values observed in fish proteins, particularly between Natterin-1 and Natterin-2, may reflect relaxed selective constraint, lineage-specific diversification or closely related proteins (Magalhaes et al., 2005). The phylogenetic separation between fungal and fish proteins, despite their overall relatedness, further supports the evolutionary diversification of these aerolysin-domain proteins across distant taxa (Moran et al., 2012). Accordingly, the low identity but moderate similarity observed for the *A. ostoyae* proteins is consistent with their classification as remote homologues of aerolysin-like domains in natterin proteins, where overall sequence divergence coexists with conservation of structurally relevant regions.

The high confidence of the ARMOST_18480 model (pLDDT > 90; pTM = 0.82), relative to the more variable scores of ARMOST_17075 and ARMOST_02021, suggests differences in predicted structural reliability, as pLDDT and pTM provide measures of local and global model confidence rather than intrinsic stability (Edmunds et al., 2024). β-barrel pore-forming proteins generally act through membrane binding, oligomerization, and subsequent conformational rearrangement to insert membrane-spanning β-strands and generate the functional pore (Kumar et al., 2023). Because the actual pore is formed by an oligomeric assembly rather than a monomer, the AGIP motif contributes to the membrane-insertion and oligomerization steps required for pore assembly (Lima et al., 2021). In Natterin proteins, the AGIP motif has been associated with the pore-forming region, supporting a conserved structural role (Lima et al., 2021). Therefore, the consistent positioning of AGIP-like motifs within the predicted pore-forming regions of *A. ostoyae* proteins further supports their structural relevance and potential involvement in conserved functional mechanisms (**Supplementary Table 7**).

Structural comparisons showed low similarity to the canonical aerolysin fold (TM-score ~0.25-0.31), suggesting substantial topological divergence, whereas moderate similarity to natterin proteins (TM-score ~0.40-0.50) suggests closer structural affinity within a divergent aerolysin-like architecture (Xu and Zhang, 2010). This observation is consistent with the general principle that protein structures can remain conserved despite substantial sequence divergence, a phenomenon well documented in pore-forming toxin families where distinct sequences give rise to similar membrane-active architectures (Mondal et al., 2020). Together, these results support the classification of these proteins as structurally conserved but evolutionarily divergent aerolysin-like proteins.

Several limitations of the present study should be acknowledged. All functional inferences are based on computational predictions; domain annotation, structural modeling, secretion prediction, and transcriptomic profiling and have not been validated by biochemical or cell biological experiments. The pore-forming activity of the identified proteins has not been demonstrated *in vitro*, and their capacity to disrupt lipid bilayers, lyse cells, or interact with specific glycan receptors remains to be tested.

Importantly, the conserved structural framework of aerolysin-like proteins is directly linked to their pore-forming function, which underlies their diverse biological and applied potential. Their affinity for cell membranes (Anton et al., 2025) enables applications such as biosensors (Podobnik et al., 2017), as well as antimicrobial and antiviral strategies with relatively low cytotoxic effects (De Cena et al., 2024). For instance, Cressiot et al. (2019) highlighted that aerolysin can be adapted into a highly sensitive nanopore sensor for single-molecule detection and biomolecular analysis, with applications including DNA sequence discrimination and biomarker detection at very low concentrations. For example, this approach can distinguish oligonucleotide sequences and identify disease-related molecules at very low concentrations. In addition, aerolysin exhibits high stability across a wide range of conditions, remaining functional at temperatures up to ~70 °C, across broad pH ranges, and in the presence of denaturing agents, which supports its suitability for robust analytical and biotechnological applications. Besides this, aerolysin has also been applied in the selective isolation of parasites such as *Trypanosoma* and *Leishmania* from blood or tissue samples. These organisms are resistant to the toxin, whereas host blood cells are susceptible, enabling targeted lysis of mammalian cells and subsequent parasite purification (Alouf et al., 2015).

The structural tractability of these proteins in the present study may contribute to the future engineering of aerolysin-like proteins with potential anticancer applications, similarly to experimental targeted cancer therapy approaches using engineered BinB toxins (Kumkoon et al., 2023). In this context, the high-confidence ARMOST_18480 model highlights their potential as scaffolds for protein engineering, especially within the N-terminal regions, which may exhibit increased flexibility or reduced evolutionary constraint.

## Data Availability

The original contributions presented in the study are included in the article/**Supplementary Material**, further inquiries can be directed to the corresponding author/s.

## Supplementary material

Supplementary Figure 1 Schematic representation of conserved domain organization in candidate aerolysin-like proteins identified in *Armillaria ostoyae* and *Armillaria mellea*. **(A)** Proteins containing ricin B-type lectin and aerolysin-like domains. **(B)** Proteins containing fascin-like and aerolysin-like domains. **(C)** Proteins containing only aerolysin-like pore-forming domains.

Supplementary Figure 2 Maximum likelihood phylogenetic analysis of selected *Armillaria ostoyae* aerolysin-like proteins and representative natterin proteins.

Supplementary Figure 3 *De novo* motif discovery analysis of aerolysin-like proteins and representative natterin homologs using MEME Suite.

Supplementary Figure 4 Predicted aligned error (PAE) plots of AlphaFold3 structural models for the candidate *Armillaria ostoyae* aerolysin-like proteins ARMOST_18480, ARMOST_17075, and ARMOST_02021. Darker green regions indicate higher confidence in relative residue positioning, whereas lighter regions represent increased positional uncertainty between protein regions.
